# Authors’ Reply to: Techniques to Teach Students Effectively Using Telemedicine. Comment on “Incorporating Medical Students Into Primary Care Telehealth Visits: Tutorial”

**DOI:** 10.2196/37401

**Published:** 2022-03-11

**Authors:** Aanika Balaji, Sarah Lou Clever

**Affiliations:** 1 Johns Hopkins University School of Medicine Baltimore, MD United States; 2 Department of Medicine Johns Hopkins University Baltimore, MD United States

**Keywords:** medical student, education, primary care, telehealth, video visits, internal medicine, medical education, teleconsultation, digital health, COVID-19, teaching, telemedicine, clerkships

We appreciate the comments by Kandola and Minhas [1] on our paper [2] and their perspective on telemedicine as senior medical students. We wanted to comment on the additional recommendations the authors suggested.

**See One, Do One, Teach One**

We agree with the approach of first observing visits to understand the flow and format; then conducting a visit with the preceptor in the room for immediate feedback and support if needed; and finally, conducting visits independently, then presenting an assessment and plan to the preceptor outside the room. Importantly, discussing the flow prior to the clinic day sets expectations, allows the student to prepare appropriately, and permits for structured feedback to be given [3,4]. As described by Dornan et al [3], the student can progress through passive observation to active observation to participation to appropriate independence. Teleclinics are perfect opportunities for students to practice and advance through each of these stages.

**Early Patient Calls**

In this approach, the student would call patients in the morning and formulate a concise history, assessment, and plan. Some considerations for this model are whether patients are available in the morning. Patients often choose telemedicine appointments to reduce travel time and fit in appointments between busy work schedules [5,6]. These patients may not be amenable to two encounters for one visit. However, permission could be established prior to students contacting the patients.

An additional consideration is whether telemedicine clinics should mirror outpatient clinics. In an in-person clinic, the student would see patients independently and quickly formulate their thoughts to present to their preceptors during each patient visit. The immediate feedback from the preceptor is lost in this telemedicine clinic format. However, calling patients early then presenting these batched visits to the preceptor later could be used at the start of a telemedicine rotation. This way, students have more time with each patient early on and can aim to transition into the telemedicine clinic with their preceptor to conduct the first portion of the visit.

As medical institutions are becoming more comfortable with and adept at telemedicine, there are multiple successful ways to engage learners. With the increased use of telemedicine, it is imperative learners are exposed to this platform to deliver care early in their training.
